# The English Education Exhibition

**Published:** 1900-01-13

**Authors:** 


### THE ENGLISH EDUCATION EXHIBITION.

THE exhibition of the chief implements of English education, which was opened by the Prince of Wales on Friday, at the Imperial Institute, and which has been brought together in order to allow of a selection being made from it for the forthcoming great exhibition at Paris, is one which should prove attractive from various points of view, and not least in its relation to both the bodily and the mental health of the English people. But, after all, an exhibition which can only be of the appliances of education, and which can show nothing of the manner in which they are applied, is not altogether unlike that brick which one of the friends of our childhood extracted from the wall of the house which he desired to sell, and which he displayed in the market-place to possible purchasers as something from which they could judge of the qualities and conveniences of the building. The general results attained in a school will bear a less definite relation to the appliances themselves than to the spirit in which they are employed; and this, in the majority of instances, would either elude exhibition entirely, or would only be exhibited in the conduct of the pupils when they were brought into touch with the affairs and the realities of life. A few appliances, perhaps, would admit of profitable comparison with one another, especially with regard to points of difference which hinder, or, perhaps, facilitate, the actual performance of scholastic work. Among such appliances would be the materials used for writing and for demonstrating to classes, the paper, the pens, the black boards, or white boards, as the case may be, which are used in the course of class lessons, and which may be of very various kinds and qualities, the character of script which is encouraged or permitted, whether this be sloping or upright, and the bodily positions which are either enforced by authority or rendered necessary by the construction and proportions of the desks and seats. More important still would be a display of models to scale, showing the cubic space given to each pupil, the ventilating arrangements, the proportion of windows to wall in the class-rooms, the positions of the windows in relation to the seats, and the area of sky space which was visible from each seat. Sufficient lighting and sufficient air supply are of such importance to the efficiency of a school as almost to justify a proposal that they should be subject to the control of some licensing body other than a School Board, just as stability of structure and sufficient outlets are secured for places of public entertainment by the authority of County Councils or other powers. We have as yet only thirty years' experience of the effects of subjecting the whole of the juvenile population of the country to school influences; and the time, which has not been long enough to justify the formation of definite conclusions as to the effects of these influences upon the development of intelligence or the formation of character, has been quite sufficient to afford ample proof of the operation of schools in promoting the diffusion, although perhaps not the fatality, of epidemic diseases, and in throwing a strain which, if not actively injurious, is at least severe, upon immature portions of the organism. The natural protection of young animals against over-strain is provided by their restlessness, by the volatility with which, if uncontrolled, they pass from one pursuit or one amusement to another; and, when this restlessness and this volatility are brought under the grip of discipline, it is one of the clear necessities of the case that, unless health is to be sacrificed or imperilled, the discipline itself must be controlled by a due consideration of the needs and the weaknesses of the immature or undeveloped tissues. It follows that the school in the present day presents to the physician a series of problems of the highest interest to science, and of the most supreme importance to the community; and it is chiefly in relation to these problems that such an exhibition as that of which we write is to be valued. The exhibition shows, indeed, only the superficial aspects of education, and casts but little light, even if any, upon the deeper influences which the word is intended to connote; but it nevertheless indicates the existence of these deeper influences to all thoughtful minds, and can hardly fail to excite reflection and to stimulate inquiry with regard to the true nature of the processes to which we are subjecting the future population of our country.

